# The changing spectrum of microbial aetiology of respiratory tract infections in hospitalized patients before and during the COVID-19 pandemic

**DOI:** 10.1186/s12879-022-07732-5

**Published:** 2022-09-30

**Authors:** Sondre Serigstad, Dagfinn L. Markussen, Christian Ritz, Marit H. Ebbesen, Siri T. Knoop, Øyvind Kommedal, Lars Heggelund, Elling Ulvestad, Rune O. Bjørneklett, Harleen M. S. Grewal, the CAPNOR study group, Tristan W. Clark, Daniel Faurholt-Jepsen, Pernille Ravn, Cornelis H. van Werkhoven

**Affiliations:** 1 Emergency Care Clinic, Haukeland University Hospital, 5021 Bergen, Norway; 2 Department of Clinical Medicine, University of Bergen, 5021 Bergen, Norway; 3 Department of Clinical Science, Bergen Integrated Diagnostic Stewardship Cluster, University of Bergen, 5021 Bergen, Norway; 4 National Institute of Public Health, University of Southern Denmark, 1455 Copenhagen, Denmark; 5 Department of Microbiology, Haukeland University Hospital, 5021 Bergen, Norway; 6 Department of Internal Medicine, Vestre Viken Hospital Trust, 3004 Drammen, Norway

**Keywords:** COVID-19, SARS-CoV-2, Respiratory tract infections, Community acquired pneumonia, FilmArray pneumonia panel, Epidemiology, Molecular testing

## Abstract

**Background:**

The COVID-19 pandemic was met with strict containment measures. We hypothesized that societal infection control measures would impact the number of hospital admissions for respiratory tract infections, as well as, the spectrum of pathogens detected in patients with suspected community acquired pneumonia (CAP).

**Methods:**

This study is based on aggregated surveillance data from electronic health records of patients admitted to the hospitals in Bergen Hospital Trust from January 2017 through June 2021, as well as, two prospective studies of patients with suspected CAP conducted prior to and during the COVID-19 pandemic (pre-COVID cohort versus COVID cohort, respectively). In the prospective cohorts, microbiological detections were ascertained by comprehensive PCR-testing in lower respiratory tract specimens. Mann–Whitney’s U test was used to analyse continuous variables. Fisher’s exact test was used for analysing categorical data. The number of admissions before and during the outbreak of SARS-CoV-2 was compared using two-sample t-tests on logarithmic transformed values.

**Results:**

Admissions for respiratory tract infections declined after the outbreak of SARS-CoV-2 (p < 0.001). The pre-COVID and the COVID cohorts comprised 96 and 80 patients, respectively. The proportion of viruses detected in the COVID cohort was significantly lower compared with the pre-COVID cohort [21% vs 36%, difference of 14%, 95% CI 4% to 26%; p = 0.012], and the proportion of bacterial- and viral co-detections was less than half in the COVID cohort compared with the pre-COVID cohort (19% vs 45%, difference of 26%, 95% CI 13% to 41%; p < 0.001). The proportion of bacteria detected was similar (p = 0.162), however, a difference in the bacterial spectrum was observed in the two cohorts. *Haemophilus influenzae* was the most frequent bacterial detection in both cohorts, followed by *Streptococcus pneumoniae* in the pre-COVID and *Staphylococcus aureus* in the COVID cohort.

**Conclusion:**

During the first year of the COVID-19 pandemic, the number of admissions with pneumonia and the microbiological detections in patients with suspected CAP, differed from the preceding year. This suggests that infection control measures related to COVID-19 restrictions have an overall and specific impact on respiratory tract infections, beyond reducing the spread of SARS-CoV-2.

**Supplementary Information:**

The online version contains supplementary material available at 10.1186/s12879-022-07732-5.

## Introduction

Community acquired pneumonia (CAP) is a leading cause of hospital admissions and mortality in all age groups and most parts of the world [1–3]. Our understanding of CAP has evolved in the last years. The introduction of PCR-based methods for detecting viruses and bacteria in respiratory specimens has shown a large proportion of bacterial-/viral coinfections and pure viral infections [4–6]. Several studies have demonstrated a close interaction between different viral and bacterial pathogens, especially for coinfections with *Streptococcus pneumoniae* and influenza virus [7–10].

The global pandemic of SARS-Coronavirus-2 (SARS-CoV-2) with corresponding coronavirus disease 2019 (COVID-19) was met with different containment strategies within countries, to reduce the spread of the virus. Most European countries, including Norway, introduced during March 2020 strict public infection control measures. These included imposing social distancing, prohibiting social gatherings, use of face masks, encouraging work from home solutions, increased border control and the closure of kindergartens, schools, and universities. The extent of lockdown implemented during the outbreak of COVID-19 is unprecedented. Recent studies have shown that such measures not only decreased the transmission of SARS-CoV-2, but also contributed to a massive reduction of circulating seasonal viruses [11–13]. We hypothesized that societal infection control measures would impact the number of hospital admissions for respiratory tract infections (RTIs), as well as, the spectrum of pathogens detected in patients with suspected CAP. Thus, we analysed both aggregated patient admission data (2017–2021) to compute numbers admitted with respiratory symptoms to the emergency department (ED), and furthermore studied the detection rates for common respiratory pathogens in two cohorts with acute community acquired RTIs, recruited before and during the COVID-19 pandemic at our tertiary care hospital. To our knowledge, this is the first study to both analyse admission data and systematically compare microbiological detections by syndromic PCR-based testing of lower respiratory tract samples in adult patients hospitalized with community acquired RTIs admitted before and during the outbreak of SARS-CoV-2.

## Methods

### Patients and study design

This study consists of a retrospective study of aggregated patient data for patients admitted to the hospitals in Bergen Hospital Trust from January 2017 through June 2021, as well as, patients with suspected CAP included from two prospective cohort studies.

### Retrospective evaluation of hospital admissions

Using information from electronic health records, we captured information on the total number of admissions to the ED per month from January 2017 to June 2021, for the hospitals in Bergen Hospital Trust, including the number of patients with acute respiratory tract infections belonging to the five defined subgroups of International Classification of Diseases 10th revision (ICD-10) diagnostic codes: upper respiratory tract infections (J00–06 and J36); infections with influenza (J09–J11) other lower respiratory tract infections (J12 and J16–22); bacterial pneumonia (J13–J15); and obstructive lung diseases (J44–J46).

### Prospective cohort studies

Data were selected from two studies conducted in two consecutive winter seasons (2019/2020 and 2020/2021) at Haukeland University Hospital, a tertiary care referral centre in Bergen, Norway. The first from a cohort study with prospectively recruited patients with suspected CAP, enrolled between December 2nd 2019 and February 17th 2020 (pre-COVID cohort) [14]. The second from a cohort of prospectively enrolled patients with suspected CAP from an ongoing randomized controlled trial (RCT) (NCT04660084), recruited between September 25th 2020 and May 31st 2021 (COVID cohort). Patients with suspected CAP at admission (retrospectively diagnosed as CAP or other lower RTIs) and a specimen from the lower respiratory tract at admission were consecutively selected from the two cohorts.

The first study was conducted as a feasibility study to inform the design of the RCT from where the second patient group was selected. The RCT aims to evaluate the clinical impact of rapid diagnostic methods on antibiotic use and outcome. The inclusion- and exclusion criteria for both cohort studies were the same. Patients were eligible for inclusion if they were ≥ 18 years, presenting to the ED with a suspicion of CAP and fulfilling at least two of the following criteria: new or worsening cough; new or worsening expectoration of sputum; new or worsening dyspnoea; haemoptysis; pleuritic chest pain; radiological evidence of pneumonia; abnormalities on chest auscultation and/or percussion; fever (≥ 38.0 °C). Exclusion criteria were cystic fibrosis, severe bronchiectasis, hospitalization within the last 14 days prior to admission, a palliative approach (defined as life expectancy below 2 weeks), or if the patient was not willing or able to provide a lower respiratory tract sample.

#### *Data collection and sampling*

Patients were enrolled on weekdays between 08:00 a.m. and 09:00 p.m. Relevant baseline information was collected by study nurses or investigating physicians through a structured interview. Symptoms and findings upon clinical examinations were recorded. Data pertaining to treatment and results from laboratory tests and medical imaging were obtained from electronic medical records and charts. Data were registered in an electronic case report form (eCRF) from VieDoc™ (Viedoc Technologies, Uppsala, Sweden).

#### *Microbiological sampling and methods*

At inclusion, a lower respiratory tract sample for the BioFire® FilmArray® Pneumonia panel *plus* (FAP *plus*) (bioMérieux S.A., Marcy-l’Etoile, France) and standard culture was obtained from all patients. Depending on clinical symptoms, vital signs, and medical history, either spontaneous sputum, or sputum induced by either nebulized isotonic (0.9%) or hypertonic (5.8%) saline was collected. Patients with known obstructive lung disease and patients with hypoxemia or signs of airway obstruction upon physical examination, were additionally treated with a bronchodilator (salbutamol and/or ipratropium bromide) prior to sampling. If sputum induction was unsuccessful, endotracheal aspiration was performed.

The FAP *plus* is a commercial automated multiplex PCR panel for the detection of 27 bacteria and viruses as well as seven genetic markers of antibiotic resistance, validated for lower respiratory tract samples [15] (see Additional file 1). The standard diagnostic methods (SDs) included culture of respiratory tract samples and blood according to current guidelines (adapted from [16]). Nasopharyngeal and/or oropharyngeal swabs were examined by an in-house real-time PCR test to detect respiratory viruses and atypical bacteria. SDs also included rapid tests; the pneumococcal urine antigen test (Quidel Corporation, San Diego, US) and a point of care test (POC) for influenza virus A and B (ID NOW™, Illinois, US). The latter was only available for the pre-COVID cohort. Blood culture results deemed as contamination by the microbiologists, were not counted. Any additional tests requested by the treating physician were noted and counted as part of SDs.

### Statistical analysis

Descriptive statistics for continuous variables are reported as median with interquartile range (IQR). Mann–Whitney’s U test was used to analyse continuous variables. Fisher’s exact test was used for analysing categorical data, by use of contingency tables. The number of admissions for acute RTIs or other acute respiratory complaints before and during the outbreak of SARS-CoV-2 was compared using two-sample t-tests on logarithmic transformed values. Percentage changes with 95% confidence intervals (95% CIs) were obtained after back-transformation. A two tailed p-value ≤ 0.05 was considered statistically significant for all analyses. The statistics were performed using IBM SPSS Statistics (version 26.0; Armonk, NY, US), the statistical environment R (Vienna, Austria), GraphPad Prism (GraphPad Software, La Jolla, CA, USA) and the GraphPad QuickCalcs Web site: https://www.graphpad.com/quickcalcs/contingency1/ (last accessed 2nd May 2022).

### Ethics

The two prospective patient cohorts were selected from a study approved by the Regional Committee for Medical and Health Research Ethics in South East Norway (REK ID: 31935) and performed in accordance with the Declaration of Helsinki. Written informed consent was obtained from all participants or from their legal guardian/close relative (in case of altered consciousness or confusion) at the time of recruitment. Regarding data extracted from the electronic heath records, only aggregated anonymous patient data was used and consent was deemed unnecessary by the Regional Committee for Medical and Health Research Ethics in Western Norway (REK ID: 221336).

## Results

### Hospital admissions before and during the COVID-19 pandemic

**Fig. 1:**
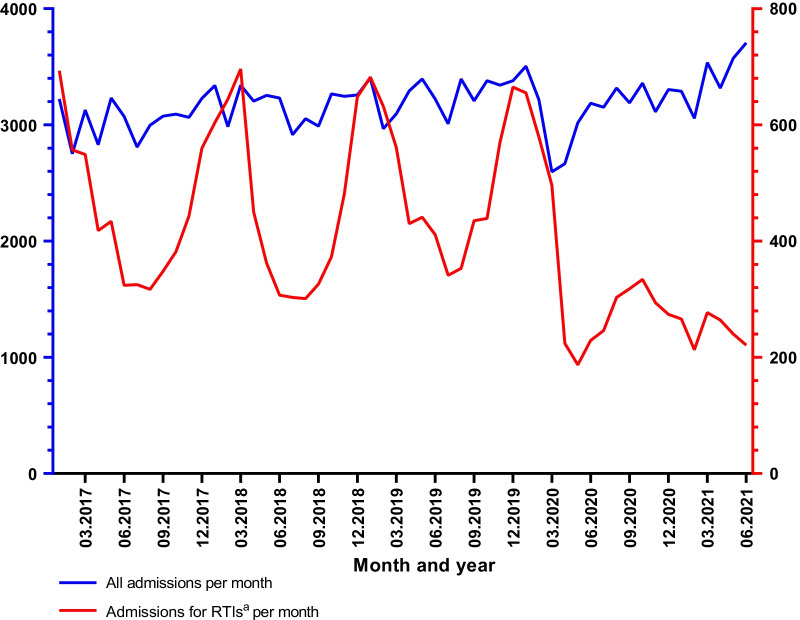
Number of visits to the emergency department. The total number of emergency department visits to the Bergen Hospital Trust per month displayed in blue. The number of patients admitted for acute RTIs^a^ is displayed in red. *RTIs* respiratory tract infections, *ICD* International Classification of Diseases. ^a^Acute RTIs or other acute respiratory symptoms defined as ICD-10 primary diagnosis of J00–06, J12–J122, J36 and J44–J46

A total of 22,870 patients were discharged with a primary diagnosis of acute RTI or other acute respiratory symptoms from January 2017 to June 2021. There were 5391 admissions in 2017, 5585 in 2018, 6108 in 2019, 4271 in 2020 and 1515 in the first 6 months of 2021. An overview of the number of visits per month to the ED in Bergen Hospital Trust is shown in Fig. 1. There was a total of 6684 admissions with upper RTIs, 1413 with influenza, 4560 with other lower RTIs (LRTIs), 5020 with bacterial pneumonia and 5193 with obstructive lung disease. The median number of admissions with RTIs and acute respiratory complaints per month was 447 (370–594) before the pandemic and 268 (236–309) after March 2020 (p < 0.001), while the total number of admissions remained stable (p = 0.672) (Table 1). The reduction in admission rate was observed for all subcategories (upper and lower RTIs, obstructive lung disease, and bacterial pneumonia) (p < 0.001). For influenza virus, 59 of the 1413 influenza cases were admitted during the COVID period, and 55 of these were admitted during March 2020.

**Table 1:** Number of admissions per month before and during the outbreak of SARS-CoV-2

Diagnosis	Jan. 2017–Febr. 2020	March 2020–June 2021	Difference (95% CI)	p-value
Upper RTI	137 (100–170)	83 (72–100)	− 37.0% (− 48.0% to − 23.6%)	< 0.001
Other lower RTI	80 (62–134)	49 (34–60)	− -46.7% (− 58.8% to − 31.2%)	< 0.001
Bacterial pneumonia	105 (94–121)	62 (53–67)	− 43.7% (− 50.1% to − 63.4%)	< 0.001
Obstructive lung disease	101 (92–116)	77 (72–87)	− 24.8% (− 32.3% to − 16.6%)	< 0.001
Total number of RTIs/acute respiratory complaints	447 (370–594)	268 (236–309)	− 41.0% (− 49.2% to − 31.5%)	< 0.001
All admissions	3219 (3053–3292)	3239 (3085–3338)	1.1% (− 4.1% to 6.6%)	0.672

Number of admissions before the COVID-19 pandemic compared with number of admissions during the COVID-19 pandemic. Numbers are given as median admissions per month with interquartile range. P-values were calculated with two sample t-tests on logarithm-transformed values. Percentage changes with 95% CIs were obtained after back-transformation

*Jan* January, *Febr* February, *95% CI* 95% confidence interval, *RTIs* respiratory tract infections

### The microbial spectrum of hospitalized RTIs before and during the COVID-19 pandemic

#### *Patient characteristics of the prospective pre-COVID and COVID cohorts*

**Fig. 2:**
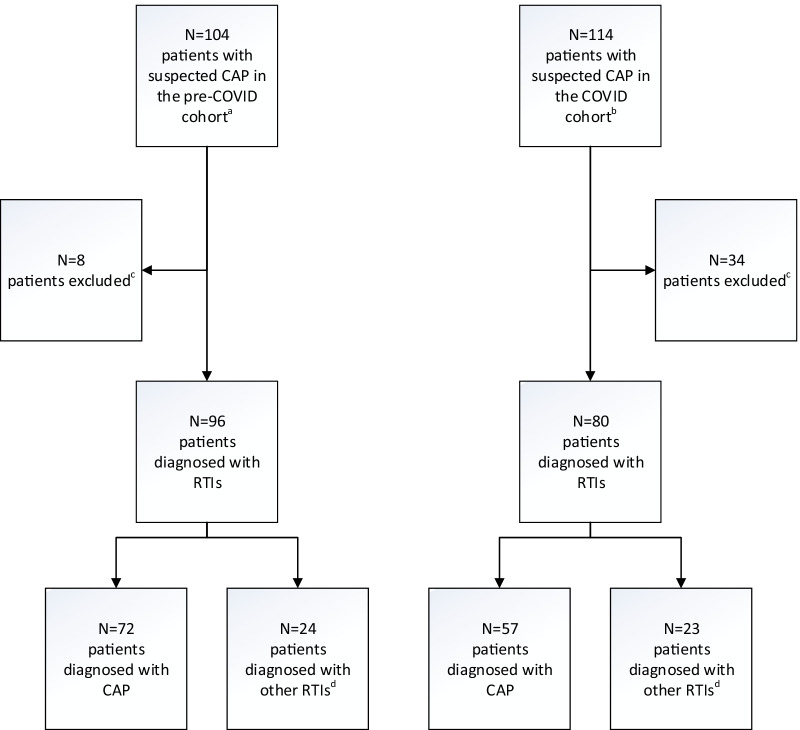
Cohort study flowchart. *CAP* community acquired pneumonia, *RTI* respiratory tract infection, *COPD* chronic obstructive pulmonary disease. ^a^Inclusion before the COVID-19 pandemic (between December 2nd 2019 and February 17th 2020). ^b^Inclusion during the COVID-19 pandemic (between September 25th 2020 and May 31st 2021). ^c^Patients were excluded due to other diagnoses, most frequently non-infectious exacerbation of COPD; heart failure; other infection; and pulmonary embolism. ^d^i.e. exacerbation of COPD/asthma other lower respiratory tract infections

A total of 176 patients with suspected CAP (i.e., confirmed CAP or other lower RTIs) were included from the two cohort studies. Ninety-six patients were from the pre-COVID cohort and included before the outbreak of SARS-CoV-2 in Norway, while 80 patients were included during the COVID-19 pandemic in Norway (COVID cohort) (Fig. 2). A lower respiratory tract specimen was obtained from all patients: 82% (144/176) by sputum induction; 9% (16/176) by spontaneous expectoration; and 9% (16/176) by endotracheal aspiration. All lower respiratory tract specimens were cultured and 95% (168/176) were also analysed by the FAP *plus*. Blood cultures were performed in 99% (175/176) of the patients, in-house PCR testing for 95% (167/176) and a pneumococcal urine antigen test for 71% (125/176) of the patients. The patient characteristics are shown in Table 2.

**Table 2:** The patient characteristics of the prospective pre-COVID and COVID cohorts (n = 176)

	Pre-COVID cohort (n = 96)	COVID cohort (n = 80)	P-value
**A: Baseline characteristics**			
*Demography*			
Age	73 (59–80)	73 (58–79)	0.966
Female	52 (54)	32 (40)	0.070
Male	44 (46)	48 (60)	0.070
*Comorbidity*			
Cardiovascular disease	48 (50)	42 (53)	0.764
Diabetes mellitus	11 (11)	11 (14)	0.655
Asthma/COPD	38 (40)	42 (53)	0.096
Kidney disease	15 (16)	7 (9)	0.252
Previous smoker	43 (45)	47 (59)	0.071
Current smoker	20 (21)	16 (20)	> 0.999
*Vaccine status*			
Influenza virus^a^	58 (60)	45 (56)	0.646
Pneumococcal	28 (29)	34 (43)	0.081
**B: Severity and outcome**			
*Severity score*^b^			
CURB-65	1.0 (1.0–2.0)	1.0 (1.0–2.0)	0.296
PSI	93 (71–111)^c^	91 (65–114)	0.864
*Outcome*			
Length of stay (days)	3.1 (2.0–5.0)	3.1 (2.0–6.1)	0.747
HDU or ICU admission	10 (10)	10 (13)	0.812
Case fatality rate			
In-hospital	1 (1)	0 (0)	> 0.999
30 days	1 (1)	1 (1)	> 0.999
60 days	4 (4)	1 (1)	0.378

Data shown as count (%) or median (IQR). P-values are calculated with Mann-Whitney’s U test and Fisher’s exact test, comparing the pre-COVID cohort with the COVID cohort

*COPD* chronic obstructive pulmonary disease, *CURB-65* confusion, urea, respiratory rate, blood pressure, age ≥ 65 years, *PSI* pneumonia severity index, *HDU* high dependency unit, *ICU* intensive care unit, *IQR* interquartile range, *CAP* community acquired pneumonia

^a^Vaccinated for influenza virus with the latest vaccine

^b^Only performed in for CAP patients

^c^Missing for five CAP patients

#### *Microbiological findings*

There was a significant decrease in detection rates for viruses in patients with suspected CAP in the COVID cohort compared with the pre-COVID cohort [21% (24/112) vs 36% (62/174), difference of 14%, 95% CI 4% to 26%; p = 0.012]. The proportion of detected bacteria remained stable between the two cohorts; 71% (79/112) in the COVID cohort vs 62% (108/174) in the pre-COVID cohort (difference of 8%, 95% CI − 3% to 20%; p = 0.162).

**Fig. 3:**
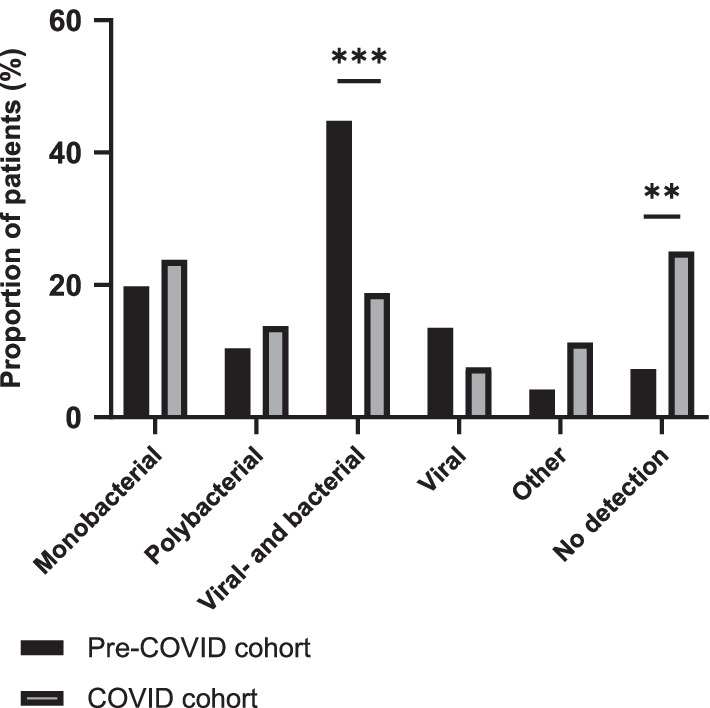
Proportion of patients stratified by microbiological detection categories. Proportion of 96 patients included before the COVID-19 pandemic (pre-COVID cohort) and 80 patients included during the COVID-19 pandemic (COVID cohort), stratified by microbiological detection categories. P-values are calculated with Fisher’s exact test. **P ≤ 0.01; ***P ≤ 0.001

Figure 3 shows the distribution of patients with viral, bacterial, and bacterial- and viral co-detections in the two cohorts. The proportion of patients with multiple detections, i.e., multiple bacteria or bacterial- and viral co-infections, was lower in the COVID cohort compared with the pre-COVID cohort [33% (26/80) vs 55% (53/96), difference of 23%, 95% CI 9% to 38%; p = 0.0037]. Indeed, the proportion of patients with bacterial- and viral co-detections in the COVID cohort was less than half compared with the pre-COVID cohort [19% (15/80) vs 45% (43/96), difference of 26%, 95% CI 13% to 41%; p = 0.0004]. Further, the number of patients with a viral detection (solely or in combination with another microbe) in the COVID cohort was also considerably lower compared with the pre-COVID cohort [26% (21/80) vs 58% (56/96), difference of 32%, 95% CI 19% to 47%; p < 0.0001].

The overview of the microbiological findings is listed in Table 3, stratified by cohort. The most frequent viral detections in the pre-COVID cohort were influenza A virus (29%) followed by human metapneumovirus (17%), while the corresponding detections in the COVID cohort were rhino-enterovirus (15%) followed by SARS-CoV-2 (13%). *Haemophilus influenzae* was the most frequent bacterial detection in both cohorts (36% and 26%, respectively). *Streptococcus pneumoniae* (25%) followed *H. influenzae* in the pre-COVID cohort, while *Staphylococcus aureus* increased significantly and was the second most common detection in the COVID cohort (23%).

**Table 3:** Overview and comparison of microbiological detections in two cohorts of patients with respiratory tract infections

Microbes	Acute respiratory tract infections			
	Pre-COVID cohort (n = 96)	COVID cohort (n = 80)	Difference in proportion (95% CI)	p-value
	Number of detections	Number of detections		
**Viruses**	62	24	–	–
Influenza A virus	29 (30)	0	− 30% (− 41% to − 20%)	< 0.0001
Human metapneumovirus	16 (17)	0	− 17% (− 26% to − 8%)	< 0.0001
Rhino-/enterovirus	3 (3)	12 (15)	12% (1% to 21%)	0.0061
Coronavirus (SARS-CoV-2)	0	10 (13)	13% (3% to 20%)	0.0003
Respiratory syncytial virus	7 (7)	0	− 7% (− 15% to 0%)	0.0164
Coronavirus (229E, OC43, HKU1, NL63)	5 (5)	0	− 5% (− 12% to 1%)	0.0641
Parainfluenza virus	2 (2)	1 (1)	− 1% (− 7% to 6%)	> 0.9999
Adenovirus	0	1 (1)	1% (− 5% to 6%)	0.4545
**Bacteria**	108	79	–	–
*H. influenzae*	35 (36)	21 (26)	− 10% (− 24% to 4%)	0.1934
*S. pneumoniae*	24 (25)	12 (15)	− 10% (− 22% to 3%)	0.1331
*S. aureus*	7 (7)	18 (23)	15% (4% to 26%)	0.0048
*M. catarrhalis*	11 (11)	8 (10)	− 1% (− 11% to 9%)	0.8114
*E. coli*	8 (8)	3 (4)	− 5% (− 13% to 4%)	0.3490
*S. agalactiae*	6 (6)	5 (6)	0% (− 9% to 8%)	> 0.9999
*P. aeruginosa*	3 (3)	2 (3)	1% (− 7% to 7%)	> 0.9999
*K. pneumoniae*	3 (3)	1 (1)	− 2% (− 8% to 5%)	0.6270
*S. marcescens*	3 (3)	1 (1)	− 2% (− 8% to 5%)	0.6270
*E. cloacae* complex	1 (1)	2 (3)	2% (− 6% to 7%)	0.5916
*Proteus* spp.	2 (2)	1 (1)	− 1% (− 7% to 6%)	> 0.9999
*K. oxytoca*	1 (1)	1 (1)	0% (− 6% to 6%)	> 0.9999
*M. pneumoniae*	2 (2)	0	− 2% (− 8% to 4%)	0.5013
*K. variicola*	0	2 (3)	3% (− 5% to 8%)	0.2052
*A. calcoaceticus*–*A. baumanii* complex	1 (1)	0	− 1% (− 6% to 5%)	> 0.9999
*L. pneumophila*	1 (1)	0	− 1% (− 6% to 5%)	> 0.9999
*S. maltophilia*	0	1 (1)	1% (− 5% to 6%)	0.4545
*S. pyogenes*	0	1 (1)	1% (− 5% to 6%)	0.4545
**Other detections:**	4	9	–	–
*C. albicans*	3 (3)	8 (10)	7% (− 3% to 15%)	0.1144
*P. jirovecii*	1 (1)	1 (1)	0% (− 6% to 6%)	> 0.9999

Microbiological detections in patients admitted with acute respiratory tract infection and who were able to provide a sample from the lower respiratory tract. Ninety-six patients from the pre-COVID cohort (included before the COVID-19 pandemic) are compared with 80 patients from the COVID cohort (included during the COVID-19 pandemic). Data are shown as counts with percent in brackets. The percentage was calculated as proportion of patients in the respective cohorts. P-values are calculated by using Fisher’s exact test

*Streptococcus pneumoniae* was detected more frequently in combination with a viral pathogen in the pre-COVID cohort compared with the COVID cohort [79% (19/24) vs 42% (5/12), difference of 38%, 95% CI 1% to 66%; p = 0.0576], moreover influenza A virus was detected in 54% (13/24) of patients with *S. pneumoniae* (pre-COVID cohort). In the COVID cohort, *S. aureus* was detected more frequently in patients with SARS-CoV-2 compared to patients without SARS-CoV-2 [60% (6/10) vs 17% (12/70), difference of 43% 95% CI 8% to 70%; p = 0.0071].

## Discussion

The Norwegian COVID-19 restrictions were comprehensive and intrusive, but comparable to other European countries [17]. During the inclusion period of our COVID cohort, strict national restrictions were still in place. This likely resulted in important lifestyle changes and an increased awareness in the general population on measures to avoid respiratory tract infections, especially in combination with continued widespread use of hand disinfection fluids and face masks. Based on retrospective surveillance of ICD-10 codes, we demonstrated a large reduction in patients admitted to our hospital with acute respiratory infections, corresponding to the period with imposed COVID-19 restrictions. The reduction was specific for acute respiratory diseases as there was no reduction in the total number of admissions. Code-based surveillance of microbiological data has several pit-falls, including physician adherence to coding-practices and an unawareness of the diagnostic repertoire performed during hospitalization. Consequently, high-quality, microbiological data cannot be collected through code-based surveillance data. This study is one of the first to compare the microbiological aetiology of lower respiratory tract infections in patients with suspected CAP included from two prospective hospital cohorts, one recruited just prior to the outbreak of the COVID-19 pandemic and the other during the pandemic. The microbiological aetiology was rigorously ascertained by a comprehensive molecular pneumonia panel combined with conventional methods. Our results confirm recent reports of substantially decreased viral detections in patients with acute respiratory tract infections after the outbreak of the COVID-19 pandemic compared to earlier years. Although, the proportion of detected bacteria remained stable in both cohorts, a difference was observed in the bacterial spectrum in the two cohorts. Further, a significant reduction in hospital admissions with acute respiratory tract infections, including bacterial pneumonia, was observed during the first year of the pandemic compared to the previous years.

The role of viruses in CAP have been increasingly recognized in the last decades. The introduction of PCR-based methods capable of rapidly and accurately detecting viral pathogens, has led to a great increase of viral detections in CAP patients, either alone or in combination with bacteria [4–6, 9, 18–20]. The high proportion of viral detections in our pre-COVID cohort, is consistent with these findings. However, the results from our COVID cohort, show a marked reduction in viral detections. Recent studies have shown a similar reduction of non-SARS-CoV-2 respiratory viruses during the pandemic compared to previous years [11–13, 21]. Specifically, the prevalence of influenza and respiratory syncytial virus (RS-virus) are shown to be almost negligible. The drop in viral detections is shown to coincide with COVID-19 control measures introduced at the start of the pandemic, and probably is a direct result of reduced person to person spread of pathogens [11–13, 21]. Interestingly, rhinoviruses showed a completely different trend with an increased rate of detection in the COVID cohort. This has also been observed in other studies, and correlates with the easing of social distancing and the reopening of schools after the summer in 2020 [22, 23].

We observed a shift in the microbial patterns of detected bacteria in the COVID cohort, including, fewer bacterial- and viral co-detections. Notably, there was a reduced proportion of patients with detected *S. pneumoniae*, *H. influenzae* or a combination of these. A large study analysing surveillance data from 26 countries and territories across six continents on invasive disease due to *S. pneumoniae*, *H. influenzae* and *Neisseria meningitidis* before and during the outbreak of SARS-CoV-2, showed a substantial and sustained reduction of hospital reported invasive disease for these pathogens [24]. Although these findings were based on all invasive diseases, we find it likely that they also reflect a decrease in respiratory infections.

The development of LRTIs, including bacterial CAP, is complex and still not completely understood. Our data indicate a reduction of detected bacterial pathogens with a known potential to transmit by respiratory droplets, like *S. pneumoniae* and *H. influenzae* after the outbreak of SARS-CoV-2. This can be interpreted in support of the hypothesis that person-to-person transmission of microbes is an important cause of LRTIs for both viruses and bacteria. However, the proportion of detected *S. aureus,* which also has the potential for person to person spread, increased in our COVID cohort. It is known that *S. aureus* tend to colonize the upper respiratory tract of adults more frequently than *S. pneumoniae* and *H. influenzae* and this might explain why *S. aureus* did not decrease in the same manner [25–29]. Nasal carriage of *S. aureus* is strongly associated with infection and clinical studies consistently describe a significantly greater risk of bacteremia among carriers [30]. Societal restrictions, as imposed during the pandemic, might therefore play a lesser role in reducing *S. aureus* invasive infections, than they do for other pathogens.

Many respiratory tract pathogens colonize the upper respiratory tract, especially among the children and elderly, without causing an infection [26–29, 31]. Any change within the host or the environment, such as a change in circulating viruses, antimicrobial treatment or reduced person-to-person spread of microbes, could potentially alter the conditions for colonization and thereby the subsequent risk of infection. We found that *S. aureus* was found more frequently in patients with detected SARS-CoV-2 compared with patients without (60% vs 17%, p = 0.0071). An interaction between viral and bacterial respiratory pathogens in CAP has been discussed for many years [7, 10, 32–37]. It is believed that a bacterial CAP was the most frequent cause of death during the influenza pandemic of 1918–19 [38]. Several reports have shown that influenza virus, by several complex interactions, can increase the potential of *S. pneumoniae* both as a colonizer and as a pathogen [10, 32, 34, 35, 37]. In our pre-COVID cohort, 79% (19/24) of all detected *S. pneumoniae* were found in combination with a viral pathogen, most frequently with influenza virus.

In relation, data from the Norwegian Cause of Death Registry, show a decrease in age-adjusted death rate of LRTIs, including CAP, in 2020 compared with 2010–2019 [39]. This indicates that the COVID-19 restrictions and dramatic decrease in circulating viral pathogens had an effect not only on the spectrum of bacterial patterns detected, but also in reducing the overall incidence of bacterial CAP.

The major strength of our work is that microbiological data were collected from two prospective studies with an attention to detail and accuracy of lower respiratory tract sampling. Lower respiratory tract samples were collected from all patients and tested with a comprehensive multiplex molecular panel in addition to standard methods in most patients. The study has limitations; the inclusion of CAP patients at a single hospital in Norway, a limited sample size and enrolment of patients restricted to fixed hours during weekdays. We excluded patients that were unable to provide a lower respiratory tract sample, e.g. due to confusion, severe hypoxemia, need of assisted ventilation, or a non-productive cough; symptoms which are often found in patients with COVID-19. In addition, in Norway, patients with mild COVID-19 were often treated outside the hospitals at designated COVID-19 wards. Finally, concerning surveillance data from electronic health records, ICD-10 diagnoses are registered by the treating physician and the primary diagnosis may have been influenced by the pandemic. Nevertheless, national surveillance data show that influenza, invasive pneumococcal disease, and systemic disease caused by *H. influenzae* have decreased [40].

In conclusion, admissions with pneumonia and microbiological detections in patients with suspected CAP during the first year of the COVID-19 pandemic, differed from the previous year, suggesting that infection control measures related to COVID-19 restrictions are effective beyond reducing the spread of SARS-CoV-2. The number of detected viruses declined, and accordingly, the proportion of patients with bacterial- and viral co-detections decreased. Furthermore, we observed a change in both the proportion and pattern of certain bacterial detections, implying that presence of viruses may facilitate colonization and infection by certain types of bacteria and play an important role in the etiopathogenesis of CAP.

## Supplementary Information

**Additional file 1.** BioFire® FilmArray® Pneumonia panel *plus* (FAP *plus*) targets.

## Data Availability

The datasets used and analysed during the current study are available from the corresponding author on reasonable request.

## Abbreviations

CAP: Community acquired pneumonia
PCR: Polymerase chain reaction
SARS-CoV-2: SARS-Coronavirus-2
COVID-19: Coronavirus disease 2019
RTI: Respiratory tract infection
ED: Emergency department
ICD-10: International Classification of Diseases 10th revision
RCT: Randomized controlled trial
eCRF: Electronic case report form
FAP *plus*: BioFire® FilmArray® Pneumonia panel *plus*
SDs: Standard diagnostic methods
IQR: Interquartile range
95% CI: 95% confidence interval
LRTIs: Lower respiratory tract infections
