# Supplementary material for: The changing spectrum of microbial aetiology of respiratory tract infections in hospitalized patients before and during the COVID-19 pandemic
Source: BMC Infect Dis. 2022 Sep 30;22:763. doi: 10.1186/s12879-022-07732-5 (PMC9523652; doi:10.1186/s12879-022-07732-5)
Supplement: Supplementary file 1 — Additional file 1. BioFire® FilmArray® Pneumonia panel plus (FAP plus) targets. [file 12879_2022_7732_MOESM1_ESM.pdf]

# Additional file 1

| BioFire® FilmArray® Pneumonia panel *plus* (FAP *plus*) targets |
|---|
| |
| **Bacteria (semi-quantitative)** |
| *Acinetobacter calcoaceticus-baumannii complex*<br>*Enterobacter cloacae*<br>*Escherichia coli*<br>*Haemophilus influenzae*<br>*Klebsiella aerogenes*<br>*Klebsiella oxytoca*<br>*Klebsiella pneumoniae group*<br>*Moraxella catarrhalis*<br>*Proteus spp.*<br>*Pseudomonas aeruginosa*<br>*Serratia marcescens*<br>*Staphylococcus aureus*<br>*Streptococcus agalactiae*<br>*Streptococcus pneumoniae*<br>*Streptococcus pyogenes* |
| **Atypical bacteria (qualitative)** |
| *Legionella pneumophila*<br>*Mycoplasma pneumoniae*<br>*Chlamydia pneumoniae* |
| **Viruses (qualitative)** |
| Influenza A virus<br>Influenza B virus<br>Adenovirus<br>Coronavirus (229E, OC43, HKU1, NL63)<br>Parainfluenza virus 1-4<br>Respiratory syncytial virus<br>Human rhinovirus/enterovirus<br>Human metapneumovirus<br>Middle East respiratory syndrome coronavirus |
| **Antibiotic resistance genes** |
| **ESBL:**<br>- CTX-M<br>**Carbapenemases:**<br>- KPC<br>- NDM<br>- OXA-48-like<br>- VIM<br>- IMP<br>**Methicilin Resistance:**<br>- mecA/mecC and MREJ |
